# Immunogenicity and protective efficacy of the HC009 mRNA vaccine against SARS-CoV-2

**DOI:** 10.3389/fimmu.2024.1416375

**Published:** 2024-07-26

**Authors:** Juan Liu, Huafeng Han, Binbin Yang, Naifang Zhang, Jing Li, Xicheng Chen, Jie Wu, Yingying Zhao, Yongsheng Yang

**Affiliations:** Nucleic Acid Medicine Innovation Center, Zhejiang Haichang Biotech Co., Ltd., Hangzhou, Zhejiang, China

**Keywords:** mRNA vaccine, SARS-CoV-2, immunogenicity, prime-boost, humoral immunity, immune protection

## Abstract

With the rapid global spread of COVID-19 and the continuous emergence of variants, there is an urgent need to develop safe and effective vaccines. Here, we developed a novel mRNA vaccine, HC009, based on new formulation by the QTsome delivery platform. Immunogenicity results showed that the prime-boost immunization strategy with HC009 was able to induce robust and durable humoral immunity, as well as Th1-biased cellular responses in rodents or non-human primates (NHPs). After further challenge with live SARS-CoV-2 virus, HC009 provided adequate protection against virus infection in hACE2 transgenic mice. Therefore, HC009 could provide significant immune protection against SARS-CoV-2.

## Introduction

Severe acute respiratory syndrome coronavirus 2 (SARS-CoV-2) has caused more than 3 million confirmed deaths, according to the World Health Organization, prompting emergency use authorization of prophylactic vaccines (1, 2).

SARS-CoV-2 belongs to the coronavirus family and is a single-stranded RNA virus. The genome of the virion includes the spike (S) protein, nucleoprotein (N), envelope (E) protein, membrane (M) protein, and other nonstructured proteins (3, 4). The S protein is a key target for virus-neutralizing antibodies (5, 6) and is commonly used as a major candidate for vaccine development, including adenoviral vector, DNA, RNA, and subunit vaccines (3, 7–10). Laboratory studies have demonstrated that S mRNA-based vaccines can induce potent and broad protective immune responses in animal models (5, 11, 12).

Additionally, human clinical phase trial results showed that mRNA vaccines are safe and effective. Currently, two commercial vaccines—mRNA-1273 (Spikevax, Moderna—NIAID) and BNT162b2 (Comirnaty, Pfizer—BioNTech)—have been granted emergency use authorization in several countries (13, 14). These vaccines are based on nucleoside-modified messenger RNA (mRNA) encoding the SARS-CoV-2 S antigen encased by a lipid nanoparticle (LNP). Human clinical phase III trial results demonstrated nearly 95% protection from developing COVID-19 (3, 15–18).

The LVRNA009 mRNA vaccine from AIM Vaccine, administered at three dose levels, could induce strong humoral and cellular immune responses, with seropositive conversion rates reaching 100% (19). Similarly, the ARCoV vaccine from Suzhou Abogen induced high concentrations of neutralizing antibodies against live SARS-CoV-2, with seroconversion rates for the live SARS-CoV-2 neutralizing antibody at 95%, accompanied by strong SARS-CoV-2-specific T-cell responses (20). Overall, the success of human clinical trials implies that the mRNA vaccines developed by a new vaccine technology have become a reality for protection against SARS-CoV-2 infections.

QTsome, a quaternary amine-based cationic lipid combined with a tertiary amine-based ionizable lipid, forms a novel LNP platform for drug delivery (21, 22). Some reports have demonstrated that the original QTsome was able to deliver oligonucleotides (21, 22). The emerging LNP platforms from Moderna, Pfizer/BioNTech, Alnylam Pharmaceuticals, and others have disclosed that their similar QTsome could also effectively deliver mRNA (23, 24).

In our study, the composition of HC009 against SARS-CoV-2 consists of mRNA encoding the full length of SARS-CoV-2 spike protein, with residues 986 and 987 substituted with proline (5, 25–27) as a protective antigen. This mRNA is encapsulated into LNPsusing 1,2-dioleoyloxy-3-trimethylammonium-propane chlorideQ12 (DOTAP·Cl, a cationic lipid), an ionizable lipid, 1,2-distearoyl-sn-glycero-3-phosphocholine (DSPC), cholesterol, and so on. This vaccine was developed by Haichang Biotech through the QTsome delivery platform, which is rapid and cost-effective.

In this paper, we assessed the protective efficacy and immunogenicity of this candidate vaccine in mice, rats, and non-human primates (NHPs). Our results demonstrated that HC009 can induce the neutralizing antibodies and spike-specific T-cell responses against the SARS-CoV-2 wild-type (WT) strain, as well as the Delta and Omicron BA.1 variants in mice, rats, or NHPs. Furthermore, HC009 significantly reduces viral RNA loads and prevents lung lesions induced by SARS-CoV-2 WT strain infection in hACE2 transgenic mice.

## Materials and methods

### Cells, viruses, and animals

Human embryonic kidney 293 cells (HEK293 cells) (ATCC CRL-3216) and HEK293T/hACE2-TMPRSS2 cells (SinoBiological, OEC003, China) were maintained in Dulbecco’s Modified Eagle’s Medium (DMEM, Gibco, 11965092, USA) supplemented with 10% fetal bovine serum (FBS, Gibco, 11965092, USA) and penicillin (100 U/mL)–streptomycin (100 mg/mL) (Gibco, 15140148, USA). SARS-CoV-2 (2019-nCoV) WT (SinoBiological, PSV001, China) or Omicron (B1.1.529) (SinoBiological, PSV016, China) or Delta (B.1.617.2) spike pseudovirus (SinoBiological, PSV011, China) was propagated in HEK293T/hACE2-TMPRSS2 cells. The SARS-CoV-2 virus (WIV04, NCBI reference sequence: NC_045512.2) separated by the Wuhan Institute of Virology was propagated in Vero E6-TMPRSS2 cells (BPS Bioscience, 78081, USA). All experiments involving SARS-CoV-2 infection were performed under Biosafety Level 3 facilities. SPF-grade 6- to 8-week-old female BALB/c mice and Sprague Dawley (SD) rats were purchased from Hunan SJA Laboratory Animal Co., Ltd. Three- to 5-year-old male rhesus macaques were purchased from Hubei Topgene Biotechnology. H11-K18-hACE2 C57BL/6JGpt mice were purchased from GemPharmatech; all animals were housed and bred under suitable temperature and humidity conditions. All procedures utilized in the study were in accordance with *Guide for the Care and Use of Laboratory Animals*. An animal care and use application for this study was reviewed and approved by TOPGENE Biotechnology IACUC (IACUC No. 2022–054 for mice, 2022–055 for rats, and 2022–059 for rhesus macaques) and Wuhan Institute of Biological Products Co., Ltd. IACUC (IACUC No. WIBP-AII442021007 for H11-K18-hACE2 C57BL/6JGpt mice).

### mRNA synthesis and encapsulation

The open reading frame (ORF) of each mRNA was inserted between the 5′ and 3′ untranslated regions (UTRs) and had a 110-base poly(A) at the 3′ end. Nucleoside-modified mRNA was produced by *in vitro* transcription (IVT) by substitution of uridine triphosphate (UTP) with N1-methylpseudouridine triphosphate (Glycogene, PU-1002, China). IVT reactions were performed using linearized DNA templates, an optimized T7 RNA polymerase mix (HZYMES biotech, HBP000330, China), NTPs, and Cap GAG m7G (5′)ppp (5′)(2′-OMeA)pG (Glycogene, CA-1005, China) and purified using an oligo-dT affinity column (Thermo Fisher Scientific, A48352, USA) and Tangential Flow Filtration (TFF, Repligen Corporation, USA). RNA integrity was analyzed by microfluidic capillary electrophoresis (Fragment Analyzer systems 5200, Agilent), and the concentration, pH, and endotoxin levels were also determined.

To prepare the mRNA vaccines, purified mRNAs were encapsulated in LNPs according to a modified procedure wherein the ionizable lipid, the cationic lipid DOTAP·Cl, DSPC, cholesterol, and so on were dissolved in ethanol at a molar ratio. The lipid and aqueous phases were mixed at a volume ratio of 1:3 with a designed micro-channel device to formulate LNPs. The LNPs were dialyzed in phosphate-buffered saline (pH 7.4) to remove ethanol for 24 h and concentrated to a desired concentration using a TFF membrane (Repligen Corporation, USA). Finally, the LNPs were passed through a 0.22-μm filter and stored at 4°C until use. Analytical characterization of the product was carried out, including the determination of particle size, polydispersity, zeta potential, and encapsulation.

### mRNA/LNP transfection

The mRNA or plasmid pAB401 (a positive control) was transfected into 1 × 10^6^ HEK293 cells in a six-well plate with the Lipofectamine 2000 transfection reagent (Thermo Fisher Scientific, 11668030, USA) according to the manufacturer’s instructions. In brief, 2.5 μg of mRNA was diluted in Opti-MEM medium (Gibco, 31985070, USA), co-incubated with 10 μL of the transfection reagent at room temperature for 20 min, and added to cells in a six-well plate. The cells were then incubated for 24 h at 37°C. In contrast, mRNA-LNPs were added directly to cells cultured with DMEM containing 10% FBS, and the cells were harvested 24 h after transfection.

### Flow cytometry

HEK293 cells were then collected and incubated with 5 μg/mL human biotinylated hACE2 protein (Sino Biological, 10108-H08H-B, China) and then with 10 μg/mL PE-Streptavidin antibody (Thermo Fisher Scientific, 12–4317-87, USA) for 20 min at room temperature. Flow cytometric (CytoFLEX, Beckman, USA) analysis of PE-positive cells confirmed protein expression.

### Western blot

HEK293 cells were harvested and lysed immediately in RIPA buffer (Beyotime, P0013B, China). An equal volume of each sample was separated by sodium dodecyl sulfate polyacrylamide gel electrophoresis, and the protein bands were transferred onto polyvinylidene fluoride membranes (Millipore, ISEQ00010). The membranes were incubated with primary antibody (rabbit polyclonal antibodies against SARS-CoV-2 spike at a dilution of 1:2,000, Sino Biological, 40589-T62, China) for 1.5 h and then with the secondary goat anti-rabbit IgG–horseradish peroxidase (HRP) (at a dilution of 1:5,000, Thermo Fisher Scientific, 31460, USA) for 45 min and visualized using a SuperSignal West Femto Substrate Trial Kit (Thermo Fisher Scientific, 34096, USA).

### Immunization and challenge

Female BALB/c mice or SD rats aged 6–8 weeks or male 3- to 5-year-old rhesus macaques were selected for the immunological evaluation of HC009. Groups of mice (*n* = 5), rats (*n* = 7), or macaques (*n* = 3) were intramuscularly immunized twice with HC009 (0.5 μg per mouse, 2 μg per mouse, or 10 μg per mouse; 30 μg per rat/macaque or 90 μg per rat/macaque) at 3-week intervals (day 1, D1 and day 22, D22). Spleen or peripheral blood mononuclear cell (PBMC) samples were acquired at the indicated time points after vaccination for enzyme-linked immunospot assay (ELISpot), intracellular cytokine staining (ICS) assay, and cytometric bead array (CBA). Serum samples were collected immediately, inactivated at 56°C for 30 min, and stored at −20°C for enzyme-linked immunosorbent assay (ELISA).

For virus challenge experiments, each group consisted of 6- to 8-week-old female H11-K18-hACE2 C57BL/6JGpt transgenic mice (*n* = 12). The mice were intramuscularly immunized twice with 0.5 μg, 2 μg, or 10 μg of HC009 at 3-week intervals. They were then intranasally inoculated with 1 × 10^3^ cell culture infective dose 50% (CCID_50_) of live SARS-CoV-2 at 50 days post-vaccination, and their body weights were monitored for 6 days. Lung and nasal turbinate tissues were harvested for viral load detection over 6 days after the challenge, and the animals were euthanized with a low dose of isoflurane.

During immunization and challenge experiments, the clinical status and food intake of the mice were monitored and recorded daily.

### ELISA

Serum-specific antibody levels were tested in mice, rats, and NHPs using an S1/S2 IgG antibody ELISA kit according to the manufacturer’s instructions (RUIXIN Biotech, RXD-AN000011 for mice, RXD-AN000010 for rats, and RXD-AN000009 for monkeys, China). Ninety-six-well ELISA plates (Corning, USA) were coated with the S1/S2 protein overnight at 4°C and blocked with 4% bovine serum albumin. Nine serial dilutions of the inactivated serum, starting at 1:500 (1:500, 1:5,000, 1:10,000, 1:20,000, 1:40,000, 1:80,000, 1:160,000, 1:320,000, and 1:640,000), were added to each well, and the plates were incubated with the secondary antibody IgG-HRP and developed by the addition of 300 µL of tetramethylbenzidine to each well. Finally, the reaction was stopped by adding 50 µL/well of 2 M sulfuric acid, and absorbance at 450 nm was determined with a spectrophotometer. The positive cutoff was the OD_450_ of the negative control plus 0.35.

### ELISpot assay

Animals were euthanized, and their spleens were removed under aseptic conditions. PBMCs were isolated from whole blood by standard density gradient centrifugation using Lymphoprep (Dakewe, 7211011, China) following the manufacturer’s instructions. To detect specific T lymphocyte responses, the IFN-γ/IL-4 ELISpot assay was performed using the IFN-γ/IL-4 ELISpot-plus Kit according to the manufacturer’s instructions (Mabtech, 3321–4APW-10 for the Mouse IFN-γ ELISpot-plus Kit, 3311–4APW-10 for the Mouse IL-4 ELISpot-plus Kit, 3220–4APW-10 for the Rat IFN-γ ELISpot-plus Kit, and 3421M-4AST-10 for the Monkey IFN-γ ELISpot-plus Kit, Sweden; U-CyTech, CT-128-PR2 for the Monkey IL-4 ELISpot-plus Kit, Holland). Splenocytes or PBMC (1 × 10^5^/well) suspensions and SARS-CoV-2 full-length S peptide mix (0.5 μg/mL, GenScript, China) were added to 96-well ELISpot plates precoated with IFN-γ/IL-4 antibodies, and phorbol 12-myristate 13-acetate (PMA) was added as a positive control. Cells incubated without stimulation were employed as the negative control. After incubation at 37°C for 24 h, the plates were washed with wash buffer, and biotinylated anti-mouse IFN-γ/IL-4 or anti-rat IFN-γ or anti-monkey IFN-γ/IL-4 antibody was added into each well at 37°C for 1 h, followed by the addition of streptavidin-HRP with an incubation at 37°C for 1 h. After the addition of chromogenic substrate for 5 min at room temperature, the reaction was terminated by washing with pure water for 2 min, and the plates were dried for 30 min at room temperature. Finally, the results of the ELISpot assays were evaluated using an AID ELISpot Reader Classic and analyzed by the ELISpot 7.0 iSpot software (AID, Germany).

### ICS assay

For mice, 5 × 10^5^ spleen cells were *ex vivo* restimulated with 1 μg/mL full-length S peptide mix or cell culture medium (no peptide) as a control. The cells were restimulated in the presence of GolgiPlug (BD Bioscience, 555029, USA) for 12 h at 37°C. Then, cells were stained with Fixable Viability Stain 700 (BD Bioscience, 564997, USA) for 10 min at room temperature and Ms CD16/CD32 Pure (a FC receptor block) (BD Bioscience, 553141, USA) for 10 min at room temperature. The cells were incubated with the Ms CD8a FITC (BD Bioscience, 553030, USA), Ms CD3e BV421 (BD Bioscience, 562600, USA), Ms CD45-APC-Cy7 (BD Bioscience, 557659, USA), and Ms CD4 BV605 (BD Biosciences, 563151, USA) surface markers for 15 min at room temperature. The cells were then fixed in permeabilization buffer (BD Biosciences, 554714, USA) for 20 min at room temperature and stained with Ms IFN-γ-PE (BD Biosciences, 554412, USA) and Ms IL-4-PE-Cy7 (BD Biosciences, 560699, USA) for 30 min at room temperature.

For rats, 5 × 10^5^ splenocytes or PBMCs were *ex vivo* restimulated with 1 μg/mL full-length S peptide mix or cell culture medium (no peptide) as control. The cells were incubated with GolgiPlug, incubated and stained with Fixable Viability Stain 700, and the Rat CD45 BV510 (BD Biosciences, 740140, USA), Rat CD3 BV421 (BD Biosciences, 563948, USA), Rat CD4 PE-Cy7 (BD Biosciences, 561578, USA), and Rat CD8A FITC (BD Biosciences, 561965, USA) surface markers. The cells were then fixed in permeabilization buffer and stained with Alexa 647 Rat IFN-γ (BD Biosciences, 562213, USA) and PE Rat IL-4 (BD Biosciences, 555082, USA).

For monkeys, 5 × 10^5^ splenocytes or PBMCs were treated as described for ICS in T cells of the mice. Afterwards, the cells were incubated with GolgiPlug, Fixable Viability Stain 700, and Hu BD Fc Block NALE (BD Biosciences, 564765, USA) and then the cells were stained with FITC NHP CD45 (BD Biosciences, 557803, USA), BV421 Human/NHP CD3 (BD Biosciences, 562877, USA), BV605 Human CD4 (BD Biosciences, 562843, USA), and BV650 Human CD8 (BD Biosciences, 563821, USA) surface markers. The cells were then fixed in permeabilization buffer and stained with APC Human/NHP IFN-γ (BD Biosciences, 551385, USA) and PE Human/NHP IL-4 (BD Biosciences, 551774, USA). All labeled lymphocytes were analyzed on a FACSCelesta (BD Biosciences, USA), and data were analyzed using FlowJo V10.

### CBA

A total of 5 × 10^5^ splenocytes or PBMCs were restimulated for 48 h with full-length S peptide mix (0.5 μg/mL final concentration per peptide) or cell culture medium (no peptide) as control. Concentrations of these indicated secreted cytokines in supernatants were determined using a bead-based CBA (BD Biosciences, Mouse IFN-γ Flex Set, 558296; Mouse IL-2 Flex Set, 558297; Mouse IL-4 Flex Set, 558298; Mouse TNF Flex Set, 558299; Mouse IL-5 Flex Set, 558302; Mouse IL-13 Flex Set, 558349; Rat IL-2 Flex Set, 561420; Rat IFN-γ Flex Set, 558305; Rat TNF Flex Set, 558309; Rat IL-4 Flex Set, 558307; Rat IL-1α Flex Set, 560159; Rat IL-10 Flex Set, 558306; NHP Th1/Th2 Cytokine Kit, 557800, USA) according to the manufacturer’s instructions. Briefly, for each sample and cytokine standard mixture, 50 μL of sample or standard was combined with 50 μL antibody capture spheres in buffer and incubated for 2 h at room temperature, and then 50 μL of detector PE-labeled antibody was added into the mixture. The mixture (150 μL) was subsequently incubated for 3 h at room temperature and washed to remove the unbound detector PE-labeled antibody reagent. Finally, the fluorescence was measured with a FACSCalibur (BD Biosciences, USA) and analyzed with FlowJo V10. Values below the lower limit of quantification were set to zero.

### Pseudovirus neutralization assay

A total of 3 × 10^4^ HEK293T/hACE2-TMPRSS2 cells were seeded in 96-well plates. For the WT spike pseudovirus neutralization (PsVN) assay, the serum was diluted, starting with a dilution of 1:30 for mice, 1:50 for rats, and 1:30 for monkey. For the SARS-CoV-2 (2019-nCoV), Delta (B.1.617.2), or Omicron (B1.1.529) spike PsVN assay in NHPs, the serum was diluted, starting with a dilution of 1:30 for Delta and 1:5 for Omicron. Each sample was then serially diluted three times in a 96-well plate. Serial dilutions of mouse serum samples were pre-incubated for 1 h at 37°C with SARS-CoV-2 (2019-nCoV) WT, Delta (B.1.617.2), or Omicron (B1.1.529) spike pseudovirus suspension in a 1:1 (vol/vol) ratio before transferring the mix to HEK293T/hACE2-TMPRSS2 cells. The inoculated cells were then incubated for 72 h at 37°C. Luciferase activity in the cells was detected using a Centro LB 963 microplate luminometer (Berthold Technologies, Germany). Percentage inhibition of RBD-hACE2 binding was computed using the following equation: % inhibition = 1 − (RFU with sample − RFU with negative control)/(RFU with positive control − RFU with negative control). IC_50_ (50% inhibitory concentration) was reported as the reciprocal of the highest dilution of serum that still yielded a 50% reduction in the number of positive infected cells per well compared to the mean of the no-serum pseudovirus positive control. Each serum sample dilution was tested in duplicate.

### Live SARS-CoV-2 neutralization assay

Neutralizing activity of serum containing live SARS-CoV-2 WT strain, Delta, and Omicron variants was determined by the plaque reduction neutralization assay. Briefly, heat-inactivated serum samples were subjected to twofold serial dilutions, starting at 1:8, and mixed with 100 CCID_50_ live viruses in a 1:1 (vol/vol) ratio, followed by incubation at 37°C for 2 h. Afterwards, the virus–serum mixture was added to preseeded 3 × 10^4^ Vero E6-TMPRSS2 cells and incubated at 37°C for 7 days. The dilution at which cytopathic effects (CPEs) appeared was recorded, and the 50% neutralization titer (NT_50_) was calculated based on the serum antibody titer that results in 50% inhibition of CPE.

### Hematoxylin and eosin staining

The lung tissues were fixed in 4% paraformaldehyde for 24 h and were cut into 4-µm tissue sections and stained with hematoxylin and eosin (H&E) for histopathological examination. Images were captured using a Pannoramic^®^ 250 Flash III (3D HISTECH, Hungary) and rendered using CaseViewer V2.4.0.

### Viral load measurement

Lung and nasal turbinate tissue viral loads were determined by quantitative real-time PCR (qRT-PCR). Briefly, mouse lungs and nasal turbinate tissues were homogenized, and 200 μL of supernatant was extracted using an AutoMate Express Instrument (Thermo Fisher Scientific, USA). The viral *N* gene was detected using a 2019-nCoV Detection Kit (DaAnGene, DA0623, China) for qRT-PCR on an ABI QuantStudio5 Real-Time PCR system (Thermo Fisher Scientific, USA). Viral loads are expressed as the viral lg copies/mg, and the limit of detection was 100 copies/mL. The following sequences were used: forward primer (*N*): 5′-GGGGAACTTCTCCTGCTAGAAT-3′; reverse primer (*N*): 5′-CAGACATTTTGCTCTCAAGCTG-3′; and probe: 5′-FAM-TTGCTGCTGCTTGACAGATT-TAMRA-3′.

### Statistical analysis

Statistical analysis was performed using GraphPad Prism 8.0 software (San Diego, CA, USA). No data were excluded from the analyses. Statistically significant differences between groups and treatments were determined by two-way analysis of variance (ANOVA), and those between groups were determined by one-way ANOVA with the Tukey multiple comparison test. All *p-*values < 0.05 were defined as statistically significant.

## Results

### Construct design and analysis of expressed antigen

**Figure 1:**
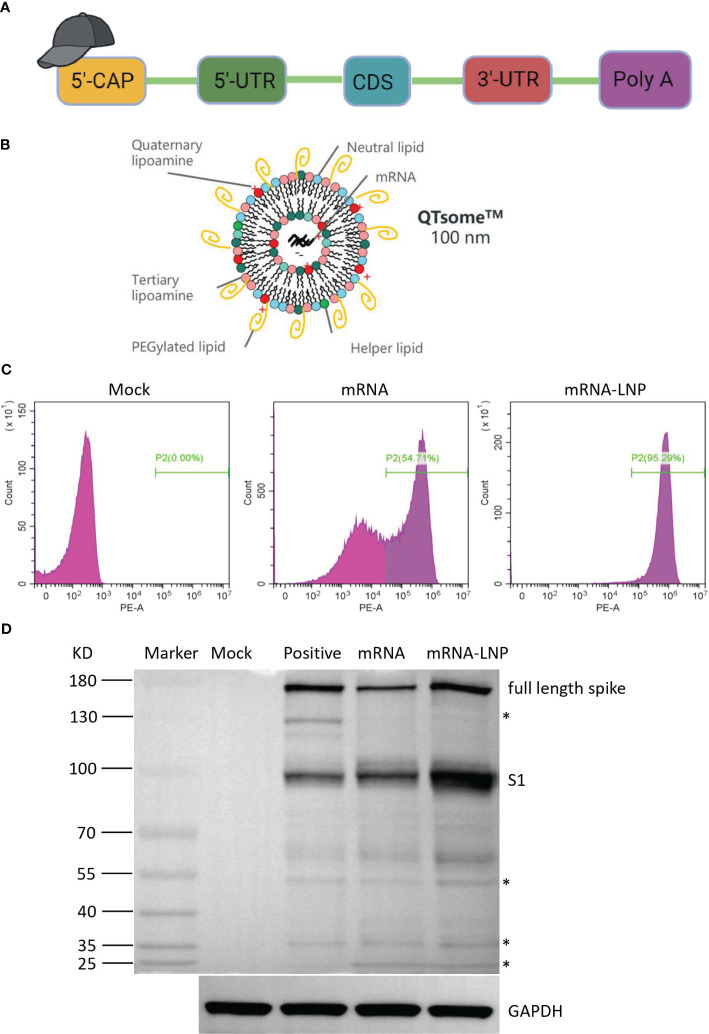
Vaccine design and expression of the mRNA vaccine encoding COVID-19 spike. **(A)** Structure of HC009 RNA. UTR, untranslated region; CDS, coding domain sequence. **(B)** Lipid nanoparticle–mRNA formulations used as COVID-19 vaccines. **(C)** Spike protein expression by flow cytometry using biotinylated hACE2 protein. **(D)** Spike protein expression analyzed by Western blot using anti-spike as the primary antibody. The asterisk indicates a non-specific band. All data are representative of three independent experiments.

The SARS-CoV-2 spike protein is the major viral antigen that induces the production of neutralizing antibodies in infected species (6). To both improve its expression and stabilize its desired conformation, we designed a panel of SARS-CoV-2 mRNA antigens. The full-length S (Wuhan-Hu-1 isolate) is stabilized in the prefusion conformation by substitution of residues 986 and 987 with proline. Briefly, the HC009 mRNA construct includes a 5′ cap structure, a 5′ UTR, an ORF, a 3′ UTR, and a poly(A) tail (**Figure 1A**). The concentration of purified mRNA was 1.42 mg/mL as measured by a NanoDrop microspectrophotometer, and the microfluidic capillary electrophoresis profile of the RNA showed a single sharp peak, indicating high purity and integrity (**Supplementary Figure S1A**). To prevent nuclease degradation and promote efficient expression *in vivo*, we encapsulated SARS-CoV-2 spike mRNA into LNPs from DOTAP·Cl, SM-102, DSPC, cholesterol, and DMG-PEG2000 at a specific ratio, forming the mRNA vaccine HC009 (**Figure 1B**). To evaluate the physical properties of the mRNA-LNPs, dynamic light scattering analyses were performed. The dynamic light scattering of LNPs revealed a mean particle size of 119.7 nm, with a narrow polydispersity index (PDI) of 0.123 and a zeta potential of 5 mV (**Supplementary Figure S1B**). The encapsulation efficiency measurement of the LNPs was 94%. Additionally, to assess the expression of mRNA or mRNA-LNPs *in vitro*, flow cytometry and Western blot results showed high expression of spike protein in HEK293 cells (**Figures 1C, D**). Therefore, all these parameters are within the acceptable criteria.

### HC009-elicited immunogenicity in mice

**Figure 2:**
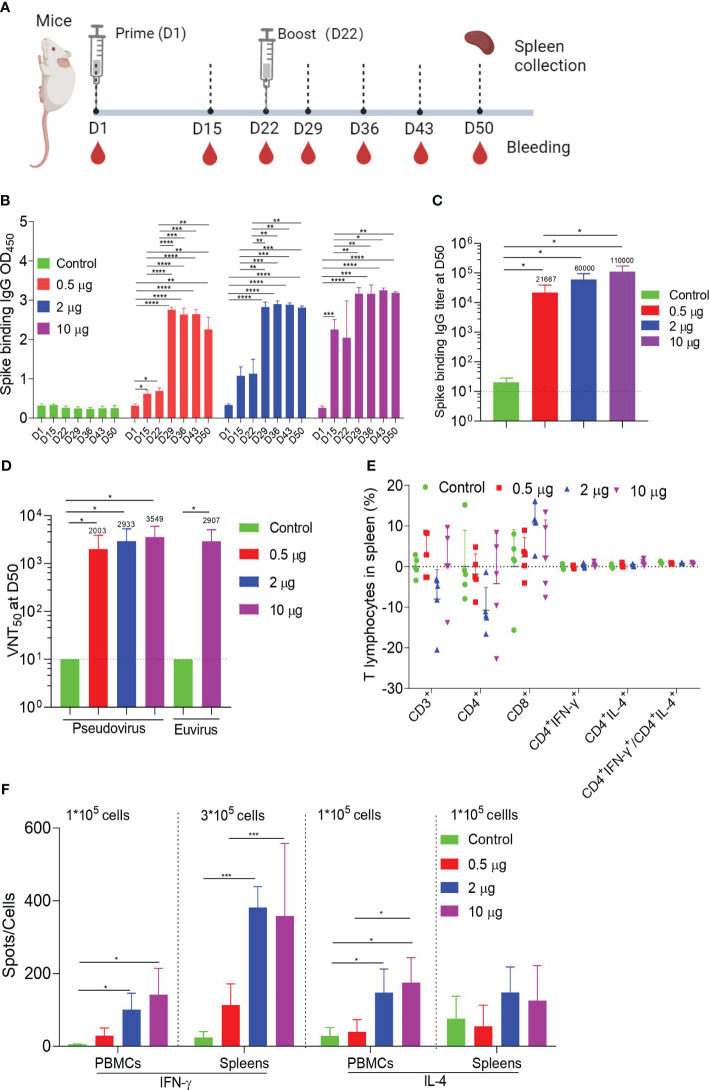
Mouse immunogenicity. **(A)** Immune procedures for evaluating HC009. Groups of 6- to 8-week-old female naive BalB/c mice (*n* = 5) were vaccinated via intramuscular injection with three doses (0.5 μg, 2 μg, and 10 μg) of HC009 at 3-week intervals. Blood collection and spleen extraction were performed at the time points shown after immunization. **(B, C)** Spike-specific IgG levels were quantified by ELISA (*n* = 5). **(D)** Neutralization assays of the SARS-CoV-2 pseudovirus and live SARS-CoV-2 were quantified (*n* = 5). **(E)** Protein-specific ICS assays. The proportions of CD3^+^, CD4^+^, CD8^+^, CD4^+^IFN-γ^+^, and CD4^+^IL-4^+^ protein-specific T cells and the ratio of CD4^+^IFN-γ^+^/CD4^+^IL-4^+^ were shown after background subtraction (*n* = 5). **(F)** Protein-specific T-cell ELISpot assay results. Splenocytes and PBMCs were stimulated with overlapping peptide pools spanning the SARS-CoV-2 full-length S protein at a final concentration of 1 μg/mL (*n* = 5). The data are shown as the mean ± standard error of the mean (SEM). Horizontal dashed line indicates the lower limit of quantification. All the data are representative of three independent experiments. One-way **(C)** or two-way **(B, D–F)** ANOVA Tukey’s multiple comparisons test was performed, *p < 0.05; **p < 0.01; ***p < 0.001; ****p < 0.0001.

To study vaccine immunogenicity, mice were immunized intramuscularly twice with doses of 0.5 μg, 2 μg, or 10 μg, or with a control, and samples were collected at specific points (**Figure 2A**). ELISA demonstrated that the spike-binding IgG OD_450_ levels induced by HC009 were significantly increasing after boosting. Compared with that at D15 or at D22 in all the dosage groups, the spike-binding IgG OD_450_ levels were significantly enhanced and were time- and dose-dependent after booster immunization (**Figure 2B**; **Supplementary Figure S2A**). Likewise, ELISA further demonstrated that the spike-binding IgG titers were significantly enhanced at D50. However, only the titers in the 10-μg group were significantly different from that in the 0.5-μg group and remained the highest among all the dosage groups (**Figure 2C**). The PsVN assay results showed that all the dosage groups elicited high neutralizing antibodies against the WT (Wuhan-Hu1) strain, compared to the control. However, no dose-dependent relationships were observed between the three dosage groups (**Figure 2D**). We also tested all the D50 samples in a live SARS-CoV-2 neutralization assay, which demonstrated strong SARS-CoV-2 neutralization activity in the 10-μg group compared to the control (**Figure 2D**). In summary, HC009 induced a high functional antibody response in mice. In the analysis of vaccine-induced T-cell responses in spleens, the distribution of the spike-specific T-cell subset (CD3^+^, CD4^+^, and CD8^+^ T cells) did not change substantially following vaccination, as assessed by flow cytometry. Similar percentages of spike-specific IFN-γ- or IL-4-producing CD4^+^ T cells, as well as the ratio of IFN-γ/IL-4-producing CD4^+^ T cells, were found in all immunized mice compared to the control (**Figure 2E**). High levels of the T-helper-1 (Th1) cytokines IFN-γ, IL-2, or TNF-α and minute levels of the T-helper-2 (Th2) cytokines IL-4, IL-5, or IL-13 were secreted, as measured in CBA. Significant differences in IFN-γ or IL-2 levels were observed between the 2-µg or 10-µg groups and the control, which indicated a Th1-driven response (**Supplementary Figure S2B**). Additionally, ELISpot results indicated that IFN-γ secretion by T lymphocytes from PBMCs or spleens was significantly higher in the 2-µg or 10-µg groups compared to the control. Notably, there were differences in IL-4 secretion by T lymphocytes from PBMCs, which were also observed in all immunized mice compared to the control (**Figure 2F**). In summary, HC009 successfully induces both humoral and Th1-biased cellular immune responses, making it an ideal candidate for SARS-CoV-2 vaccine development.

### HC009-elicited immunogenicity in rats

**Figure 3:**
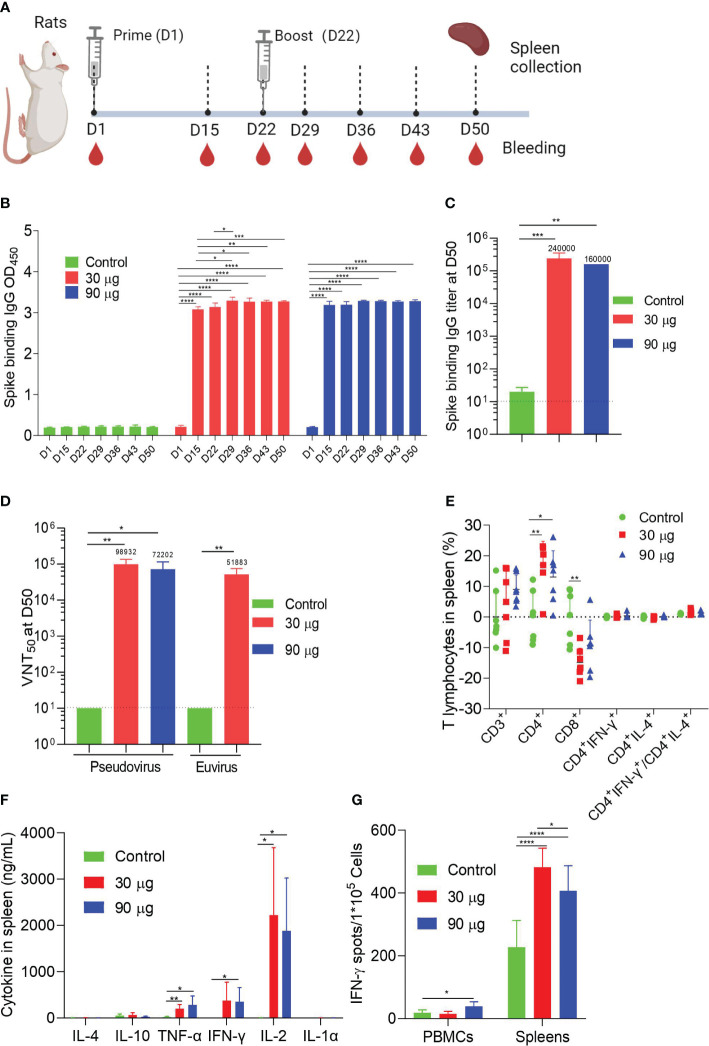
Rat immunogenicity. **(A)** Immune procedures for evaluating HC009. Groups of 6- to 8-week-old female naive SD rats (*n* = 7) were vaccinated via intramuscular injection with two doses (30 μg and 90 μg) of HC009 at 3-week intervals. Blood collection and spleen extraction were performed at the time points shown after immunization. **(B, C)** Spike-specific IgG levels were quantified by ELISA (*n* = 5). **(D)** Neutralization assays of the SARS-CoV-2 pseudovirus and live SARS-CoV-2 virus were quantified (*n* = 5). **(E)** Protein-specific ICS assays. The proportions of CD3^+^, CD4^+^, CD8^+^, CD4^+^IFN-γ^+^, and CD4^+^IL-4^+^ protein-specific T cells and the ratio of CD4^+^IFN-γ^+^/CD4^+^IL-4^+^ were shown after background subtraction (*n* = 7). **(F, G)** Protein-specific CBA **(F)** and protein-specific T-cell ELISpot assay **(G)** results. Splenocytes or PBMCs were stimulated with overlapping peptide pools spanning the SARS-CoV-2 full-length S protein at a final concentration of 1 μg/mL (*n* = 7). The data are shown as the mean ± SEM. Horizontal dashed line indicates the lower limit of quantification. All data are representative of three independent experiments. One-way **(C)** or two-way **(B, D–G)** ANOVA with Tukey’s multiple comparisons test was performed, *p < 0.05; **p < 0.01; ***p < 0.001; ****p < 0.0001.

To assess the immunogenicity of HC009 in rats, we administered intramuscular injections of either 30 μg or 90 μg of HC009 or saline control on D1 and D22. Samples were collected at specific points (**Figure 3A**). The spike-binding IgG OD_450_ levels in the immunized rats were significantly higher than those in the control after prime immunization and were further increased 7 days after boosting. The spike-binding IgG OD_450_ levels were significantly enhanced compared with that at D15 only in the 30-μg group and were time-dependent after booster immunization and dose-dependent only at D15 (**Figure 3B**; **Supplementary Figure S3A**). ELISA also further demonstrated that the spike-binding IgG titers were significantly increased at D50. Surprisingly, no apparent differences were observed between the 30-μg and 90-μg-dose groups (**Figure 3C**). Pseudovirus or live virus neutralization assays indicated that the NT_50_ levels were significantly higher in all immunized rats compared to the control, suggesting better virus neutralization activity against WT SARS-CoV-2, though no dose-dependent effect was observed (**Figure 3D**).

In addition to the humoral immunity response, ICS for cellular immunity showed that HC009 did not significantly alter the distribution of spike-specific CD3^+^, IFN-γ-producing CD4^+^, IL-4-producing CD4^+^ T cells, or the ratio of IFN-γ/IL-4-producing CD4^+^ T cells in all immunized rats, similar to the results observed in mice for splenocytes. However, a significant increase in spike-specific CD4^+^ T cells and a decrease in CD8^+^ T cells were observed in the immunized mice compared to the control (**Figure 3E**).

Regarding PBMCs, HC009 did not significantly alter the distribution of spike-specific CD3^+^, CD4^+^, CD8^+^, IFN-γ-producing CD4^+^, IL-4-producing CD4^+^ T cells, or the ratio of IFN-γ/IL-4-producing CD4^+^ T cells in all immunized rats (**Supplementary Figure S3B**). CBA results demonstrated that high levels of the cytokines IL-2, IFN-γ, or TNF-α and minute levels of the cytokines IL-4, IL-10, or IL-1α were secreted in splenocytes and PBMCs, and showed significant differences in IFN-γ, IL-2, and TNF-α levels between the 30-µg or 90-µg group and the control, which indicated a Th1-biased response (**Figure 3F**; **Supplementary Figure S3C**). Finally, ELISpot demonstrated strong IFN-γ responses after boosting in both splenocytes and PBMCs compared to the control (**Figure 3G**). In aggregate, these data indicate that HC009 strongly induces SARS-CoV-2 neutralization titers and systemic CD4^+^ T-cell Th1-biased responses in rats.

### HC009-elicited immunogenicity in rhesus macaques

**Figure 4:**
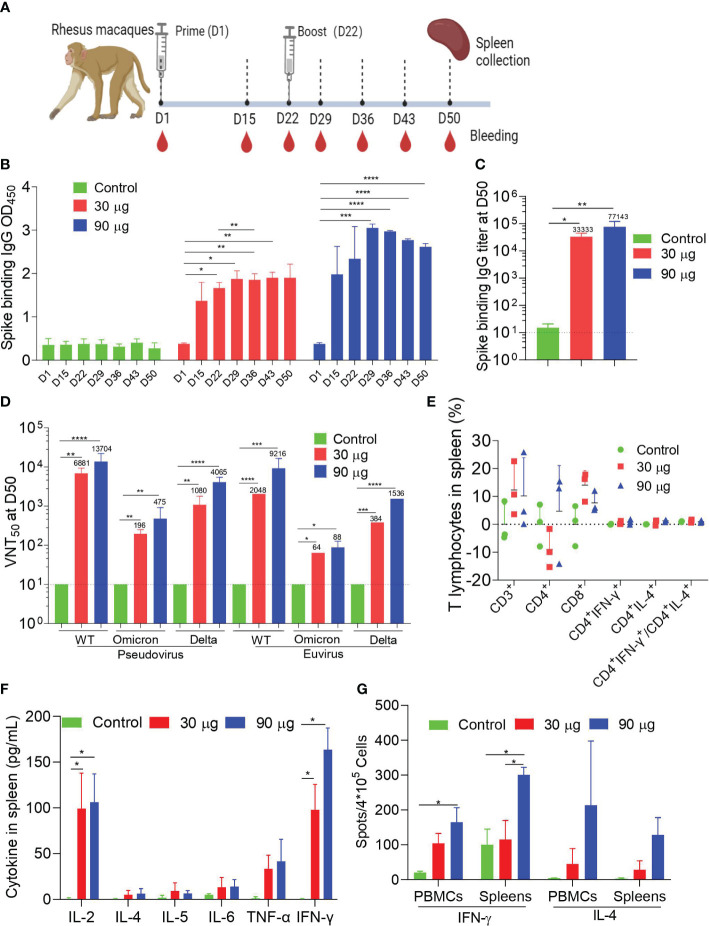
Macaque immunogenicity. **(A)** Immune procedures for evaluating HC009. Groups of 3- to 5-year-old male rhesus macaques (*n* = 3) were vaccinated via intramuscular injection with two doses (30 μg and 90 μg) of HC009 at 3-week intervals. Blood collection and spleen extraction were performed at the time points shown after immunization. **(B, C)** Spike-specific IgG levels were quantified by ELISA (*n* = 3). **(D)** Neutralization assays of the SARS-CoV-2 pseudovirus and live SARS-CoV-2 WT strain (Wuhan-Hu1), Delta (B.1.617.2), and Omicron (BA.1) variants were quantified (*n* = 3). **(E)** Protein-specific ICS assays. The proportions of CD3^+^, CD4^+^, CD8^+^, CD4^+^IFN-γ^+^, and CD4^+^IL-4^+^ protein-specific T cells, and the ratio of CD4^+^IFN-γ^+^/CD4^+^IL-4^+^ were shown after background subtraction (*n* = 3). **(F, G)** Protein-specific CBA **(F)** and protein-specific T-cell ELISpot assay **(G)** results. Splenocytes or PBMCs were stimulated with overlapping peptide pools spanning the SARS-CoV-2 full-length S protein at a final concentration of 1 μg/mL (*n* = 3). The data are shown as the mean ± SEM. Horizontal dashed line indicates the lower limit of quantification. All data are representative of three independent experiments. One-way **(C)** or two-way **(B, D–G)** ANOVA with Tukey’s multiple comparisons test was performed, *p < 0.05; **p < 0.01; ***p < 0.001; ****p < 0.0001.

To assess the immunogenicity of HC009 in nonhuman primates, we intramuscularly injected groups of three macaques (male, 2–4 years old) with 30 or 90 μg of HC009 or saline control at 3-week intervals, and samples were collected at specific points (**Figure 4A**). Prime-boost immunization of HC009 groups induced higher spike-binding IgG levels as shown by the OD_450_, compared to the control. Compared with that at D22, the spike-binding IgG OD_450_ at D36 were significantly enhanced only in the 30-μg group (**Figure 4B**). There was an obvious dose-dependent response observed between the 30-μg and 90-μg groups (**Supplementary Figure S4A**). Likewise, ELISA also further demonstrated that the spike-binding IgG titers were significantly increased with no apparent differences between these dosage groups (**Figures 4C**). The PsVN assay results further supported the immunogenicity of HC009 as it induced neutralizing antibodies against the WT (Wuhan-Hu1) strain, and Delta (B.1.617.2) and Omicron (BA.1) variants. In the 30-μg group, NT_50_ levels were highest in the WT strain, followed by the Delta variant, and weakest for the Omicron variant (**Figure 4D**). We also tested all the D50 samples in a live virus neutralization assay for the relevant WT (Wuhan-Hu1) strain, Delta (B.1.617.2), and Omicron (BA.1), which showed a similar trend of neutralizing activity to the PsVN results (**Figure 4D**). In summary, HC009 induced a strong humoral immune response in nonhuman primates.

Next, ICS confirmed that HC009 did not elicit a high distribution of spike-specific T-cell subsets, including CD3^+^, CD4^+^, CD8^+^, IFN-γ-producing CD4^+^, IL-4-producing CD4^+^ T cells, and the ratio of IFN-γ/IL-4-producing CD4^+^ T cells in all immunized animals, for both splenocytes and PBMCs, as assessed by FACS (**Figure 4E**; **Supplementary Figure S4B**). Similar results with that in mice or rats as measured in CBA in splenocytes and PBMCs showed that IFN-γ and IL-2 levels in the 30-µg or 90-µg groups were significantly increased compared with that in the control, which also indicated a Th1-driven CD4^+^ T-cell response (**Figure 4F**; **Supplementary Figure S4C**). Finally, ELISpot demonstrated strong IFN-γ but not IL-4 responses after booster immunization in splenocytes and PBMCs compared to the control (**Figure 4G**). Overall, our study demonstrates that HC009 induces robust and durable humoral and Th1-biased cellular immune response in NHPs.

### Protective efficacy of HC009 in K18-hACE2 transgenic mice

**Figure 5:**
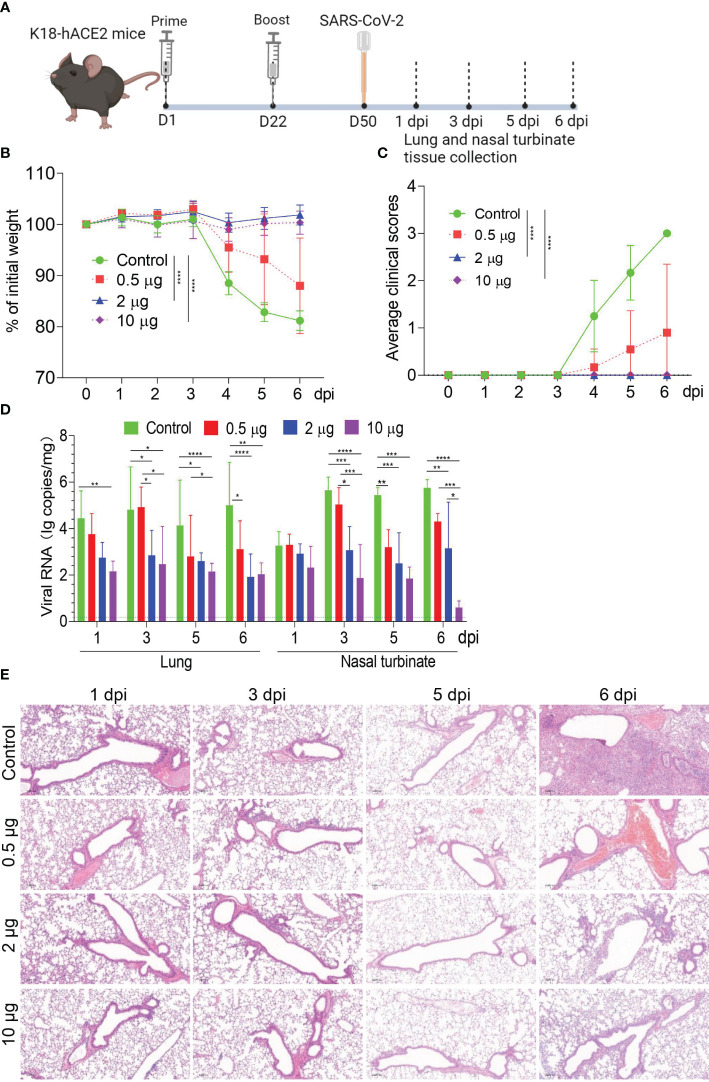
Evaluation of the immune protection provided by HC009 during *in vivo* challenge. **(A)** Immunization and challenge procedures for the 0.5-, 2-, and 10-μg dose of HC009 in mice. Six- to 8-week-old female hACE2 transgenic mice were immunized with two doses of the vaccines via the intramuscular route at 3-week intervals (*n* = 12). Subsequently, they were challenged with live SARS-CoV-2 at 50 days post-vaccination, and the lung and nasal turbinate tissues were collected at the indicated time points after immunization. **(B)** The body weights of the mice were monitored and recorded for six consecutive days after the challenge (*n* = 6). The mice were euthanized after observation. **(C)** Average clinical scores for disease signs, including lethargy, ruffled fur, hunched back posture, and rapid breathing. A score of 1 was given to each of these clinical signs (*n* = 12). **(D)** Viral RNA in the lungs and nasal turbinate tissues of challenged mice were measured with qRT-PCR at 1, 3, 5, and 6 dpi, respectively (*n* = 6). **(E)** H&E staining was performed to assess pathological changes in the lungs of mice at 1, 3, 5, and 6 dpi (*n* = 3). The data are shown as the mean ± SEM. Horizontal dashed line indicates the lower limit of quantification. All the data are representative of three independent experiments. A two-way ANOVA with Tukey’s multiple comparisons test was performed, *p < 0.05; **p < 0.01; ***p < 0.001; ****p < 0.0001.

To evaluate vaccine protection against live viruses, a lethal challenge of SARS-CoV-2 following two doses of HC009 was tested in a human ACE2 (hACE2) transgenic C57BL/6 mouse model. K18-hACE2 transgenic mice immunized with either 0.5 μg, 2 μg, or 10 μg of HC009 were intranasally challenged with a clinical isolate of SARS-CoV-2 (10^3^ CCID_50_) at day 50 post-vaccination (**Figure 5A**). Mice were then divided into two groups: one group was tracked for weight and clinical scores; a second group was euthanized at 6 days post-injection (dpi), and viral loads and histopathological evaluations were assessed in the lungs and nasal turbinate tissues. Mice that received HC009 in the 2-µg or 10-µg-dose group showed no significant change in weight and no clinical signs. In contrast, the negative control mice that received PBS or the 0.5-μg-dose group mice showed a significant drop in weight and increased clinical scores upon challenge with WT SARS-CoV-2 (**Figures 5B, C**). Consistent with the body weight and clinical sign data, both the 2-µg and 10-µg dose vaccinations could obviously reduce viral RNA load in infected lung and nasal turbinate tissues compared with unvaccinated animal controls (**Figure 5D**). To further observe the pathological changes in the lungs after infection, paraffin-embedded tissue sections were subjected to H&E staining. No significant pathological features were observed in the lungs of mice in the 2-µg and 10-µg-dose groups, while alveolar damage, inflammatory cellular infiltration, and hemorrhage were observed in the alveolar space of mice immunized with the unvaccinated animal controls (**Figure 5E**). These data demonstrate that a two-dose immunization strategy with a 2-µg dose of HC009 is sufficient to produce a protective immune response against SARS CoV-2 in mice.

## Discussion

Owing to the spread worldwide by SARS-CoV-2 variants, a safe, effective, and broad-spectrum vaccine is urgently needed. As we all know, there are two key factors that play an important role in vaccine effectiveness: one is sequence design, and the other is the drug delivery system. However, original LNPs as a drug delivery system impedes cellular uptake and release of nucleic acid cargoes into the cytoplasm, causing less efficient gene delivery than other emerging LNPs platforms (23, 24).

DOTAP·Cl is one of the original synthetic cationic lipids used for the liposomal transfection of oligonucleotides in gene therapy. The key structural element of DOTAP·Cl is its quaternary ammonium headgroup that is responsible for interactions with both nucleic acids and target cell membranes (28). It is also used in a broad range of applications, such as vaccine and anticancer drug formulations (28), as an adjuvant for immune response enhancement (29, 30). Herein, we optimized the formulation based on the LNP compositions from Moderna and Pfizer—BioNTech by adding DOTAP.Cl and an ionizable lipid to deliver a mRNA against SARS-CoV-2.

Studying the immunogenicity is a necessary step in vaccine development. So far, an increasing number of reports indicated that mRNA vaccine can induce both cellular and humoral immunity to block SARS-CoV-2 and variant infection (3, 31–33). We conducted the study of the immunogenicity in different species such as mice, rats, and monkeys for the first time. The present results demonstrated that a two-dose immunization of HC009 was able to induce a strong response of spike-specific antibodies and neutralizing antibodies against the WT SARS-CoV-2 strain, and the Delta and Omicron variants in mice, rats, and NHPs. ELISpot and ICS assays showed that HC009 could also activate SARS-CoV-2-specific T-cell immunity in mice, rats, and NHPs. In the challenge study, 2 μg of HC009 could significantly reduce the WT viral RNA loads in the lungs or nasal turbinate tissues and reduce lung histopathology in hACE2 transgenic mice. Therefore, HC009 could elicit significant humoral and cellular immune responses against SARS-CoV-2.

We have also compared the immunogenicity data and found that the immunogenicity varied among different species. For example, the titers of binding antibodies and neutralizing antibodies were the highest in rats, followed by mice, and the lowest in NHPs. Immunizing rats with the 30-µg or 90-µg dose did not elicit dose-dependent antibodies titers or IFN-γ levels, and the titer or IFN-γ levels at high doses was actually lower than that at low doses. This may be due to an excessively high dose, which could have led to the saturation of immune response (34). Beyond this threshold, increasing the vaccine dosage fails to yield a proportional increase in antibody titers or IFN-γ levels. Unexpectedly, after analysis, higher levels of induced IFN-γ were determined in the spleens compared to that in PBMCs, and this pattern was consistent across all three species while that of IL-4 was higher in PBMCs.

ICS assays showed that the proportions of spike-specific CD4^+^ T lymphocytes in the spleens of rats were significantly upregulated, while those of CD8^+^ T lymphocytes were significantly downregulated. However, the trends were reversed compared to what were observed in the spleens and PBMCs of mice or NHPs, although they were not significant, indicating that mice and NHPs are universal vaccine efficacy models, while the immunogenicity of the study in rats may not be suitable.

At present, the two mRNA vaccines are Spikevax and Comirnaty, which both express the full-length S protein, and produced pNAb titers of ~10^2–3^ at the 30-μg dose were observed in NHPs (14, 18). Unlike the current formulation used to develop SARS-CoV-2 vaccines, our vaccine formulation was specifically optimized based on the LNP compositions from Moderna and Pfizer—BioNTech by adding DOTAP.Cl and an ionizable lipid, and this formulation has been confirmed in multiple studies to produce similar or higher pNAb titers (~10^3–4^ at the 30-μg dose). Therefore, the use of low dose reduced the cost of vaccine production and gained enough immunoprotective efficacy. The vaccines of SARS-CoV and other animal coronaviruses have been reported to induce the antibody-dependent enhancement response (35, 36). Therefore, our vaccine design minimizes the effects by reducing the production of non-neutralizing antibodies.

Multivalent vaccines are already being used against different viruses and variants, and their broad-spectrum protective effects can be demonstrated (3, 37–39). Meanwhile, some reports showed that multivalent COVID-19 vaccines based on the full-length S or S1 proteins of the WT virus and Beta variant also produced a broad spectrum of neutralization titers against different viruses and variants (3, 40, 41). Surprisingly, in the paper, the cross-PsVN assays in NHPs showed that HC009 produced a broad spectrum of neutralization titers against different pseudoviruses or euvirus, but the neutralization antibody levels elicited by the vaccine against the variants were declining, especially for the Omicron lineage. Our findings were consistent with previous reports (32, 42, 43). Collectively, our regimen can potentially be used as a reference for combating SARS-CoV-2.

Previous evidence has revealed that both CD4^+^ T-cell types support antigen-specific antibody generation and maturation (5). In some animal models of respiratory virus infection, a Th2-type CD4^+^ T-cell response has caused vaccine-associated enhanced respiratory disease (5, 44, 45). Therefore, a Th1-type response to immunization is preferred as it may reduce the theoretical risk of enhanced pulmonary disease during subsequent viral infection. Here, our results showed that HC009 induced strong Th1-type-biased CD4^+^ T-cell responses in multiple models.

Our study does have some limitations. Although HC009 has shown good protection against SARS-CoV-2 in mice, its safety and efficacy have not been investigated in NHPs or rats. In addition, because of biosafety facility limitations, we are not able to obtain protective efficacy data in rats or NHPs against prevalent SARS-CoV-2 variants.

In conclusion, we demonstrated that the HC009 mRNA vaccine based on a new modified QTsome delivery system could induce robust immunogenicity in multiple animal models and protective efficacy in mice against SARS-CoV-2. Significantly, the neutralization cross-effects of HC009 against WT, Delta, and Omicron variants highlight the power of mRNA vaccines in protecting against the developing SARS-CoV-2 virus. These promising preclinical results support further clinical development for the potential application in preventing COVID-19.

## Data availability statement

The original contributions presented in the study are included in the article/**Supplementary Material**. Further inquiries can be directed to the corresponding authors.

## Ethics statement

The animal study was approved by Topgene biotechnology IACUC and Wuhan Institute of Biological Products Co., LTD IACUC. The study was conducted in accordance with the local legislation and institutional requirements.

## Author contributions

JuL: Conceptualization, Writing – original draft, Writing – review & editing, Supervision. HH: Data curation, Formal analysis, Writing – review & editing, Conceptualization. BY: Data curation, Formal analysis, Writing – review & editing, Conceptualization. NZ: Data curation, Formal analysis, Writing – review & editing. JiL: Data curation, Formal analysis, Methodology, Writing – review & editing. XC: Formal analysis, Methodology, Writing – review & editing, Data curation. JW: Writing – review & editing, Data curation, Formal analysis. YZ: Project administration, Writing – review & editing. YY: Conceptualization, Supervision, Writing – review & editing.

## Supplementary material

Supplementary Figure 1 The integrity analysis of mRNA and the physical properties of mRNA-LNP. **(A)** The mRNA of purity and integrity was detected by a Fragment Analyzer system. **(B)** The particle size, uniformity, and zeta potential of mRNA vaccine HC009 were measured by a Malvern particle size instrument. All the data are representative of three independent experiments.

Supplementary Figure 2 Mouse immunogenicity. Immune procedures for evaluating HC009. Groups of 6- to 8-week-old female naive BalB/c mice (*n* = 5) were vaccinated via intramuscular injection with three doses (0.5 μg, 2 μg, and 10 μg) of HC009 at 3-week intervals. Blood collection and spleen extraction were performed at the time points shown after immunization. **(A)** Spike-specific IgG levels were quantified by ELISA (*n* = 5). **(B)** Splenocytes were stimulated with overlapping peptide pools spanning the SARS-CoV-2 full-length S protein at a final concentration of 1 μg/mL (*n* = 5). The data are shown as the mean ± SEM. All the data are representative of three independent experiments. Two-way ANOVA Tukey’s multiple comparisons test was performed, **p* < 0.05; ***p* < 0.01; ****p* < 0.001; *****p* < 0.0001.

Supplementary Figure 3 Rat immunogenicity. **(A)** Immune procedures for evaluating HC009. Groups of 6- to 8-week-old female naive SD rats (*n* = 7) were vaccinated via intramuscular injection with two doses (30 μg and 90 μg) of HC009 at 3-week intervals. Blood collection and spleen extraction were performed at the time points shown after immunization. Spike-specific IgG levels were quantified by ELISA (*n* = 5). **(B)** Protein-specific ICS assays. The proportions of CD3^+^, CD4^+^, CD8^+^, CD4^+^IFN-γ^+^, and CD4^+^IL-4^+^ protein-specific T cells and the ratio of CD4^+^IFN-γ^+^/CD4^+^IL-4^+^ were shown after background subtraction (*n* = 7). **(C)** Protein-specific CBA results. PBMCs were stimulated with overlapping peptide pools spanning the SARS-CoV-2 full-length S protein at a final concentration of 1 μg/mL (*n* = 7). The data are shown as the mean ± SEM. All data are representative of three independent experiments. Two-way ANOVA with Tukey’s multiple comparisons test was performed, **p* < 0.05; ***p* < 0.01; ****p* < 0.001; *****p* < 0.0001.

Supplementary Figure 4 Macaque immunogenicity. Immune procedures for evaluating HC009. Groups of 3- to 5-year-old male rhesus macaques (*n* = 3) were vaccinated via intramuscular injection with two doses (30 μg and 90 μg) of HC009 at 3-week intervals. Blood collection and spleen extraction were performed at the time points shown after immunization. **(A)** Spike-specific IgG levels were quantified by ELISA (*n* = 3). **(B)** Protein-specific ICS assays. The proportions of CD3^+^, CD4^+^, CD8^+^, CD4^+^IFN-γ^+^, and CD4^+^IL-4^+^ protein-specific T cells, and the ratio of CD4^+^IFN-γ^+^/CD4^+^IL-4^+^ were shown after background subtraction (*n* = 3). **(C)** Protein-specific CBA results. PBMCs were stimulated with overlapping peptide pools spanning the SARS-CoV-2 full-length S protein at a final concentration of 1 μg/mL (*n* = 3). The data are shown as the mean ± SEM. All data are representative of three independent experiments. Two-way ANOVA with Tukey’s multiple comparisons test was performed, **p* < 0.05; ***p* < 0.01; ****p* < 0.001; *****p* < 0.0001.
