# Supplementary figures and images for: Immunogenicity and protective efficacy of the HC009 mRNA vaccine against SARS-CoV-2
Source: Front Immunol. 2024 Jul 26;15:1416375. doi: 10.3389/fimmu.2024.1416375 (PMC11310568; doi:10.3389/fimmu.2024.1416375)

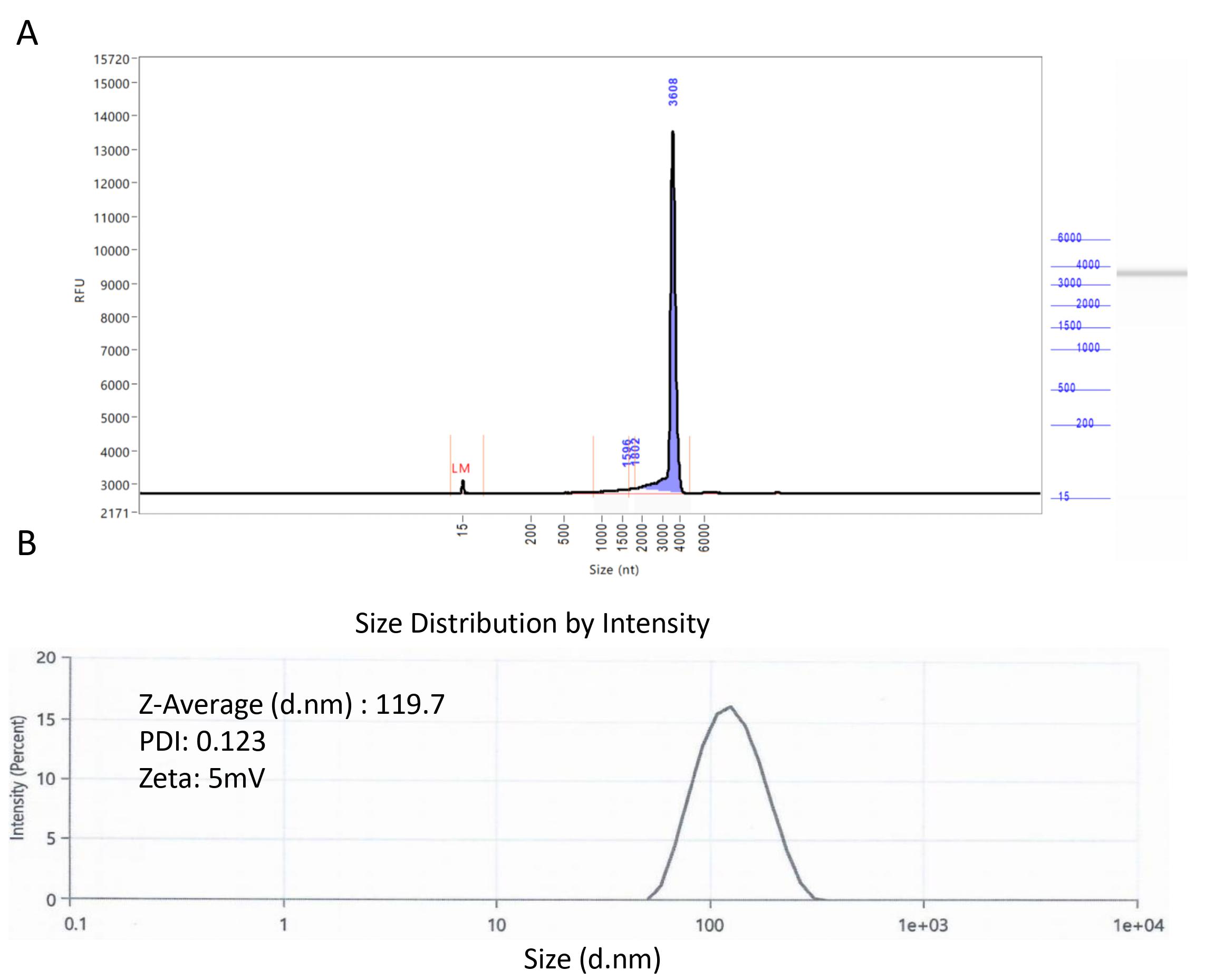

Supplement: Supplementary Figure 1 — The integrity analysis of mRNA and the physical properties of mRNA-LNP. (A) The mRNA of purity and integrity was detected by a Fragment Analyzer system. (B) The particle size, uniformity, and zeta potential of mRNA vaccine HC009 were measured by a Malvern particle size instrument. All the data are representative of three independent experiments. [file Image_1.jpeg]

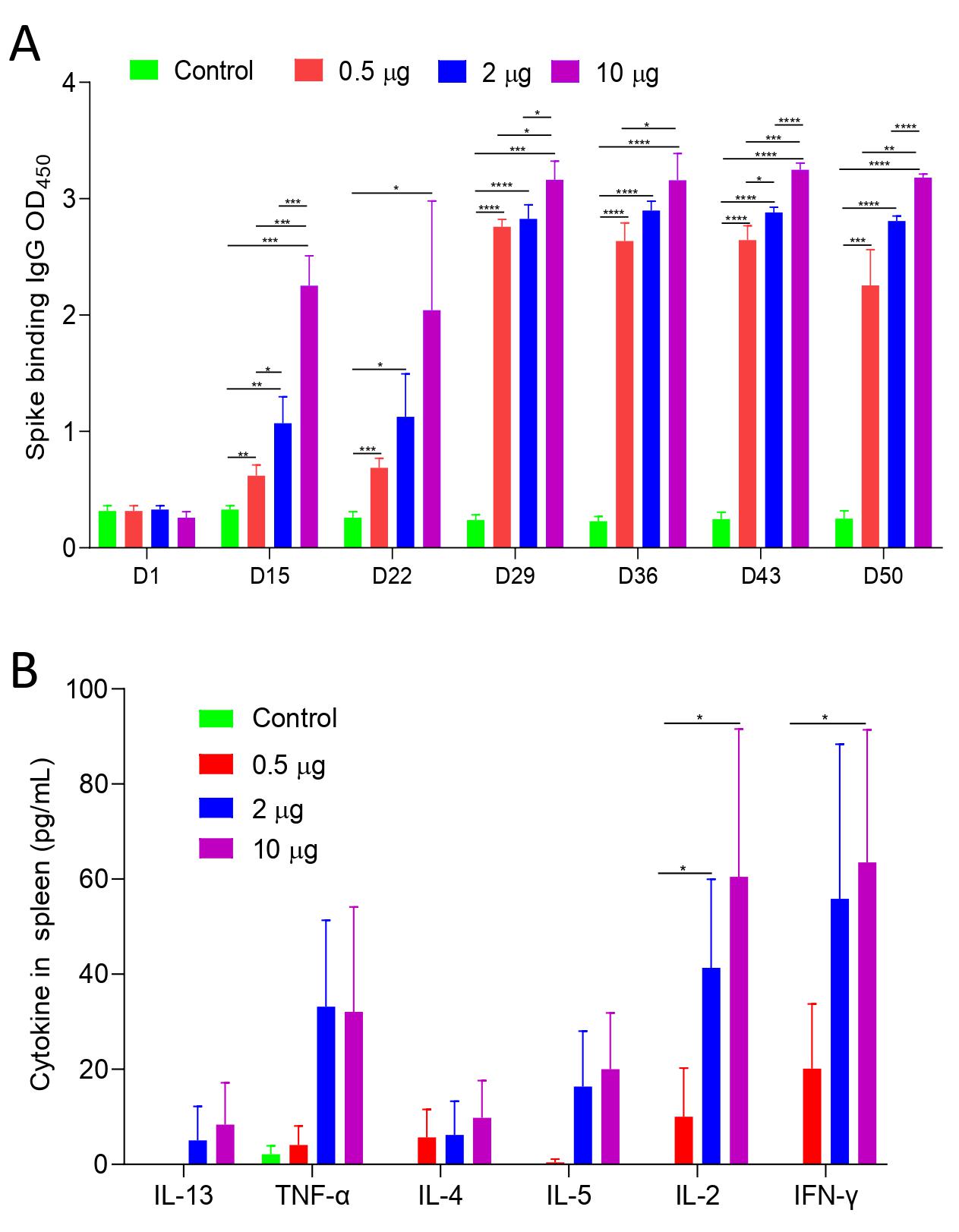

Supplement: Supplementary Figure 2 — Mouse immunogenicity. Immune procedures for evaluating HC009. Groups of 6- to 8-week-old female naive BalB/c mice (n = 5) were vaccinated via intramuscular injection with three doses (0.5 μg, 2 μg, and 10 μg) of HC009 at 3-week intervals. Blood collection and spleen extraction were performed at the time points shown after immunization. (A) Spike-specific IgG levels were quantified by ELISA (n = 5). (B) Splenocytes were stimulated with overlapping peptide pools spanning the SARS-CoV-2 full-length S protein at a final concentration of 1 μg/mL (n = 5). The data are shown as the mean ± SEM. All the data are representative of three independent experiments. Two-way ANOVA Tukey’s multiple comparisons test was performed, *p < 0.05; **p < 0.01; ***p < 0.001; ****p < 0.0001. [file Image_2.jpeg]

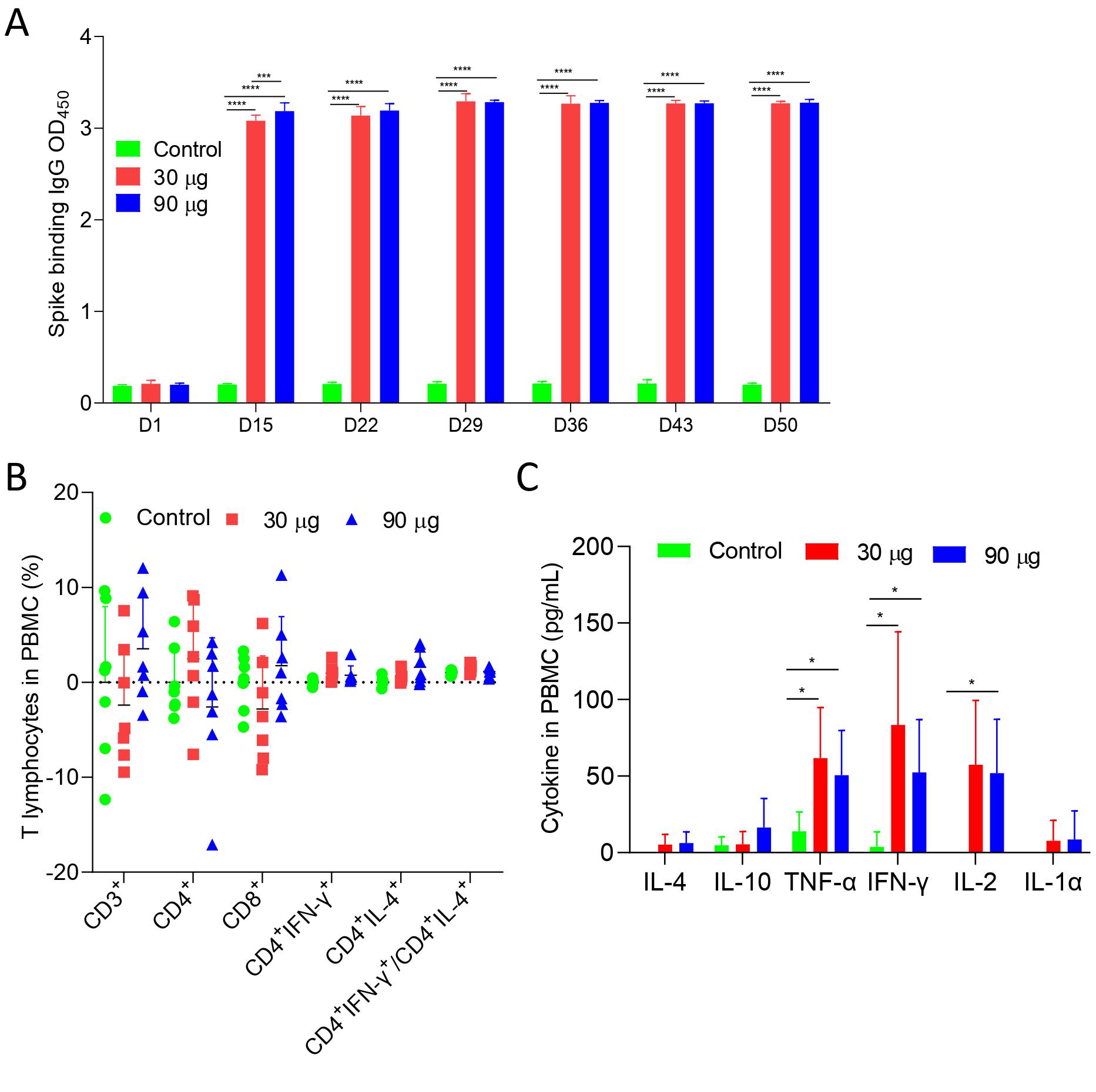

Supplement: Supplementary Figure 3 — Rat immunogenicity. (A) Immune procedures for evaluating HC009. Groups of 6- to 8-week-old female naive SD rats (n = 7) were vaccinated via intramuscular injection with two doses (30 μg and 90 μg) of HC009 at 3-week intervals. Blood collection and spleen extraction were performed at the time points shown after immunization. Spike-specific IgG levels were quantified by ELISA (n = 5). (B) Protein-specific ICS assays. The proportions of CD3+, CD4+, CD8+, CD4+IFN-γ+, and CD4+IL-4+ protein-specific T cells and the ratio of CD4+IFN-γ+/CD4+IL-4+ were shown after background subtraction (n = 7). (C) Protein-specific CBA results. PBMCs were stimulated with overlapping peptide pools spanning the SARS-CoV-2 full-length S protein at a final concentration of 1 μg/mL (n = 7). The data are shown as the mean ± SEM. All data are representative of three independent experiments. Two-way ANOVA with Tukey’s multiple comparisons test was performed, *p < 0.05; **p < 0.01; ***p < 0.001; ****p < 0.0001. [file Image_3.jpeg]

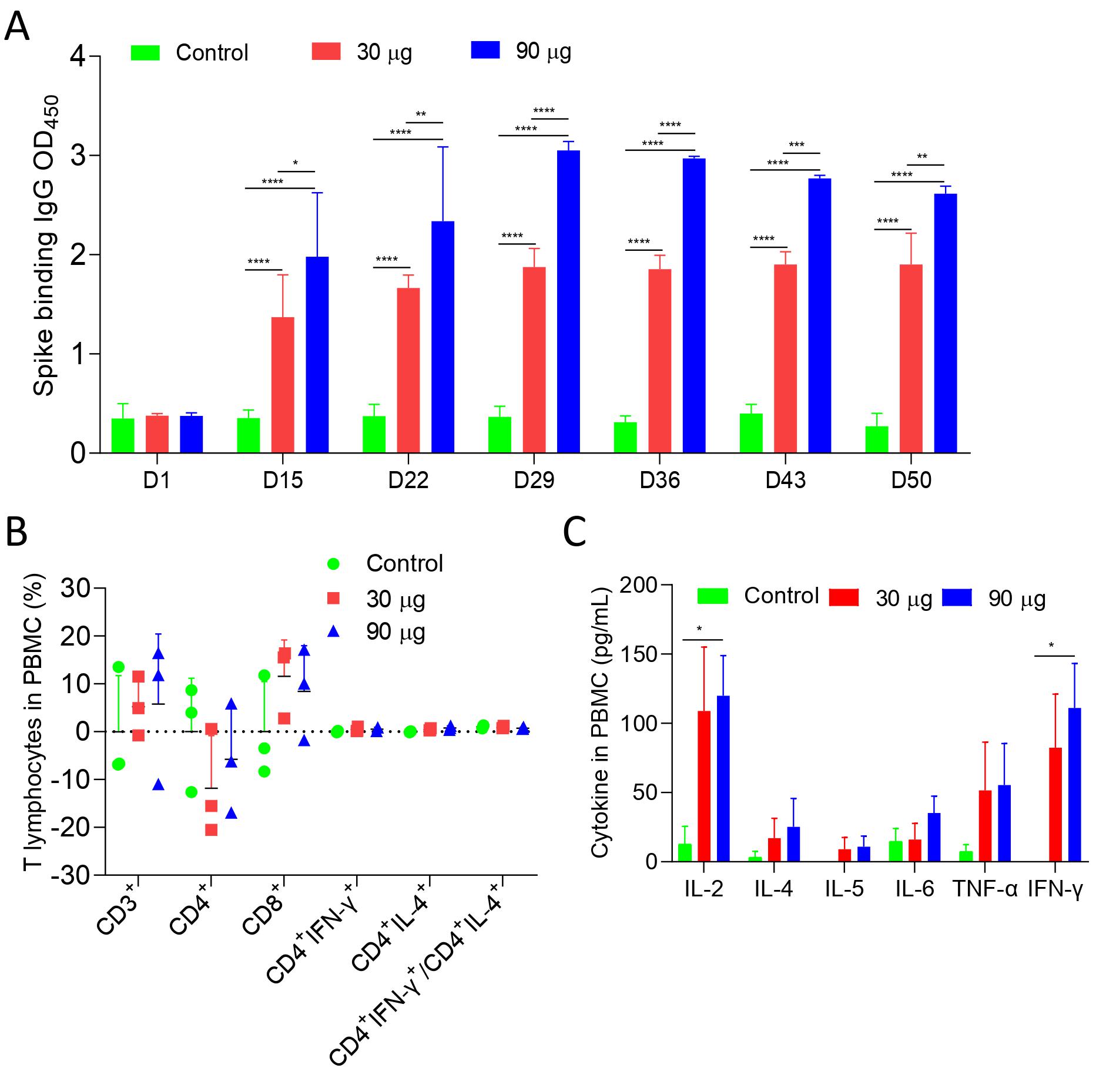

Supplement: Supplementary Figure 4 — Macaque immunogenicity. Immune procedures for evaluating HC009. Groups of 3- to 5-year-old male rhesus macaques (n = 3) were vaccinated via intramuscular injection with two doses (30 μg and 90 μg) of HC009 at 3-week intervals. Blood collection and spleen extraction were performed at the time points shown after immunization. (A) Spike-specific IgG levels were quantified by ELISA (n = 3). (B) Protein-specific ICS assays. The proportions of CD3+, CD4+, CD8+, CD4+IFN-γ+, and CD4+IL-4+ protein-specific T cells, and the ratio of CD4+IFN-γ+/CD4+IL-4+ were shown after background subtraction (n = 3). (C) Protein-specific CBA results. PBMCs were stimulated with overlapping peptide pools spanning the SARS-CoV-2 full-length S protein at a final concentration of 1 μg/mL (n = 3). The data are shown as the mean ± SEM. All data are representative of three independent experiments. Two-way ANOVA with Tukey’s multiple comparisons test was performed, *p < 0.05; **p < 0.01; ***p < 0.001; ****p < 0.0001. [file Image_4.jpeg]
